# Effects of magnetic resonance-guided high-intensity focused ultrasound ablation on bone mechanical properties and modeling

**DOI:** 10.1186/s40349-015-0033-8

**Published:** 2015-08-11

**Authors:** Sin Yuin Yeo, Andrés J. Arias Moreno, Bert van Rietbergen, Natalie D. ter Hoeve, Paul J. van Diest, Holger Grüll

**Affiliations:** Department of Biomedical Engineering, Eindhoven University of Technology, High Tech Campus 11-p1.243, 5656 AE Eindhoven, The Netherlands; Department of Pathology, University Medical Center Utrecht, Room H04.312, Utrecht, The Netherlands; Philips Research Europe, High Tech Campus 11-p1.261A, 5656 AE Eindhoven, The Netherlands

**Keywords:** HIFU, Focused ultrasound, Bone, Ablation, Bone mechanical properties

## Abstract

**Background:**

Magnetic resonance-guided high-intensity focused ultrasound (MR-HIFU) is a promising technique for palliative treatment of bone pain. In this study, the effects of MR-HIFU ablation on bone mechanics and modeling were investigated.

**Methods:**

A total of 12 healthy rat femurs were ablated using 10 W for 46 ± 4 s per sonication with 4 sonications for each femur. At 7 days after treatments, all animals underwent MR and single photon emission computed tomography/computed tomography (SPECT/CT) imaging. Then, six animals were euthanized. At 1 month following ablations, the remaining six animals were scanned again with MR and SPECT/CT prior to euthanization. Thereafter, both the HIFU-treated and contralateral control bones of three animals from each time interval were processed for histology, whereas the remaining bones were subjected to micro-CT (μCT), three-point bending tests, and micro-finite element (micro-FE) analyses.

**Results:**

At 7 days after HIFU ablations, edema formation around the treated bones coupled with bone marrow and cortical bone necrosis was observed on MRI and histological images. SPECT/CT and μCT images revealed presence of bone modeling through an increased uptake of ^99m^Tc-MDP and formation of woven bone, respectively. At 31 days after ablations, as illustrated by imaging and histology, healing of the treated bone and the surrounding soft tissue was noted, marked by decreased in amount of tissue damage, formation of scar tissue, and sub-periosteal reaction. The results of three-point bending tests showed no significant differences in elastic stiffness, ultimate load, and yield load between the HIFU-treated and contralateral control bones at 7 days and 1 month after treatments. Similarly, the elastic stiffness and Young’s moduli determined by micro-FE analyses at both time intervals were not statistically different.

**Conclusions:**

Multimodality imaging and histological data illustrated the presence of HIFU-induced bone damage at the cellular level, which activated the bone repair mechanisms. Despite that, these changes did not have a mechanical impact on the bone.

## Background

High-intensity focused ultrasound (HIFU) is a non-invasive thermal therapy, which uses acoustic energy to locally heat the tissue to ablative temperature, thereby leading to cell death [1, 2]. This technique is often performed under the guidance of magnetic resonance imaging (MR-HIFU) to aid treatment planning, enable temperature monitoring to ensure precise heating during treatments, and immediate follow-up assessments thereafter. To date, MR-HIFU has been utilized in the clinic for treatment of uterine fibroids [3–5] and is currently under investigation for treatment of prostate [6–8], breast [9, 10], liver [11, 12], and pancreatic [13, 14] cancers.

In the recent years, MR-HIFU has also been approved as an alternative palliative treatment method for cancer-induced bone pain in radiation refractory patients. This approach was assumed to alleviate pain through periosteal denervation. In patients with bone metastases, MR-HIFU ablations provided pain relief in 60–100 % of the patients [15–20]. Similarly, in patients with osteoid osteoma, complete pain relief was observed in 90 % of the patients from 1 month after treatments until the 12-month follow-up period [21]. Interestingly, the palliative efficacy of MR-HIFU ablation has been extended to treatment of osteoarthritic pain of the facet joints [22, 23] and knee [24]. In the knee osteoarthritic pain study, the visual analog scale (VAS) scores of 75 % of the patients were reduced from the third day following treatments and the observed pain relief effects remained at 6-month follow up [24]. In addition to pain palliation, preclinical [25] as well as clinical [16, 18] results have shown the prospect of HIFU-induced osteogenesis. Bucknor et al. demonstrated that MR-HIFU ablations of healthy pig femurs caused new bone formation at the treated sites [25]. In clinical studies, ablation of osteolytic lesions led to de novo mineralization of cortical bone [16, 18].

Although MR-HIFU ablation provides pain relief and promotes skeletal remodeling, this treatment method also induces cortical bone damage. As illustrated by different preclinical studies using healthy bones, osteocyte necrosis was detected in the ablated cortical bone [26–28]. Therefore, aggressive ablation of bone may compromise bone strength. Recently, the effects of MR-HIFU treatments on the mechanical properties of bone were first examined by Herman et al. in pig ribs [29]. In their study, a reduction in bone mechanical properties was observed at 6 weeks after HIFU treatments, but these changes reversed at 12 weeks after ablations. A drawback of their study is that non-weight-bearing bones were used and that ribs have a different morphology and function than long bones. As weight-bearing bones are more at risk of pathological fractures, assessing the effects of MR-HIFU on the biomechanical properties of weight-bearing bones is essential and warranted [30]. Therefore, in this study, the effects of MR-HIFU ablation on the mechanical properties of weight-bearing bones in a rat animal model were investigated by using three-point bending tests and micro-finite element (micro-FE) analyses. The animal model was chosen as it allows a full comparative study with small animal imaging, histology, and eventually tumor-bearing bones. We chose the time points of 7 days and 1 month for our study to reduce transient effects directly after HIFU treatments, while keeping it comparable to other ablation studies in small animals [31–34]. Subsequently, the findings were correlated with bone damage and modeling as assessed by multimodality imaging techniques and histological analysis.

## Methods

### Study design

**Fig. 1:**
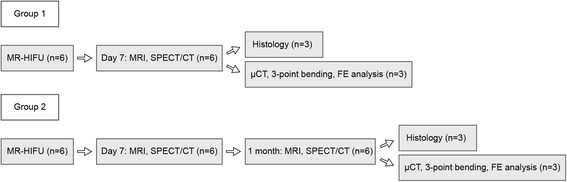
Experimental groups and their respective experimental timelines

All animal experiments were approved by the local animal welfare committee (Maastricht University, The Netherlands) and conformed to the ethical guidelines set by the institutional animal care committee. Male Copenhagen rats with a minimum age of 12 weeks were used (Jackson Laboratory, USA). Two experimental groups were included in this study with *n* = 6 for each group: (1) animals euthanized at 7 days after treatments (group 1) and (2) animals euthanized 1 month after treatments (group 2) (Fig. 1). At the beginning of the study, all animals underwent MR-HIFU ablation on their respective left femurs. At 7 days after treatments, animals from group 1 were subjected to MRI and single photon emission computed tomography/computed tomography (SPECT/CT) prior to euthanization. Thereafter, both the HIFU-treated and contralateral healthy femurs were excised. Femurs from three animals were processed for histological analyses, whereas the remaining femurs were scanned using micro-CT (μCT) for micro-FE analyses and subsequently tested in a three-point bending apparatus (Fig. 1). The animals in group 2 were scanned with MRI and SPECT/CT at 7 days and 1 month after treatments. Next, the animals were euthanized, and all femurs were processed similarly to the animals in group 1 (Fig. 1).

### MR-HIFU ablation

**Fig. 2:**
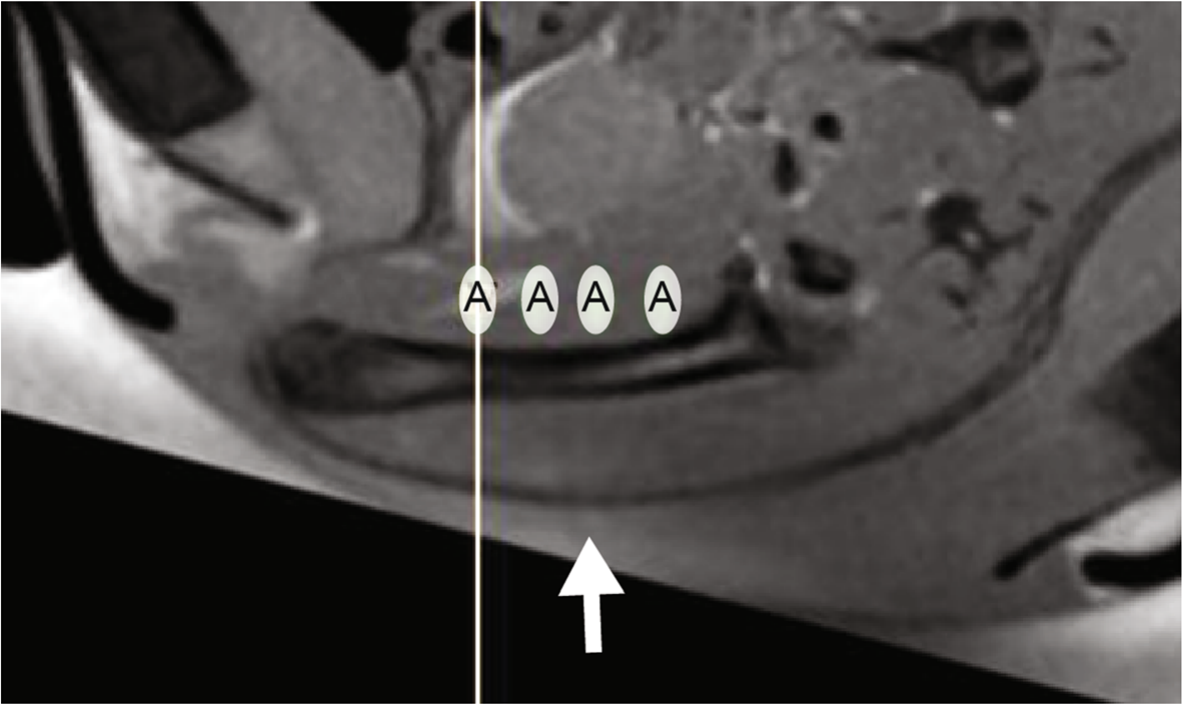
Representative T_1_-weighted image for position of treatment cells for MR-HIFU ablation. Direction of ultrasound beam (*white arrow*). Treatment cells (*A*)

Prior to HIFU ablation, the animals were given carprofen (Rimadyl®, Pfizer Inc., New York, USA) at 4 mg/kg body weight to relieve treatment-related pain. Then, the limb subjected to treatment was shaved and covered with degassed ultrasound gel (Aquasonic 100, Parker Laboratories, Fairfield, USA). Subsequently, the limb was submerged in degassed water and positioned in a multichannel small animal MR receiver coil to enable usage with a clinical 3-T MR-HIFU platform (Philips Sonalleve®, Vantaa, Finland). T_1_-weighted fast field echo (FFE, repetition time (TR) = 500 ms, echo time (TE) = 3.2 ms, field of view (FOV) = 100 × 70 × 71 mm^3^, voxel size = 0.5 × 0.5 × 1.0 mm^3^, number of signal averages (NSA) = 4) and T_2_-weighted turbo spin echo (TSE, TR = 20,752 ms, TE = 43 ms, FOV = 100 × 70 × 71 mm^3^, voxel size = 0.5 × 0.5 × 1.0 mm^3^, NSA = 2) sequences were acquired for treatment planning. Four treatment cells (2 × 2 × 7 mm^3^) were positioned behind the bone and along the femoral shaft (Fig. 2). Several sub-therapeutic sonications (acoustic frequency = 1.44 MHz, acoustic power = 5 W, duration = 20 s per sonication, continuous wave ultrasound) were performed to ensure temperature increase in the planned treatment sites. HIFU ablation was performed using 10 W acoustic power for 46 ± 4 s. During the treatment, MR thermometry sequences (RF-spoiled gradient with echo planar imaging (EPI) readout, EPI factor = 7, TR = 38 ms, TE = 20 ms, FOV = 250 × 250 mm^2^, voxel size = 1.4 × 1.4 × 4.0 mm^3^, SENSE factor = 1.8, fat suppression = spectral presaturation with inversion recovery (SPIR), NSA = 2, dynamic scan time = 4.8 s) were acquired using one slice parallel and three slices perpendicular to the ultrasound beam. Following ablation, a gadolinium-based contrast agent (Dotarem®, Guerbet LLC, Bloomington, USA) was injected at 0.2 mmol/kg body weight and contrast-enhanced (CE)-MR images were acquired using a T_1_-weighted FFE sequence for assessment of non-perfused regions. T_2_-weighted images were acquired for assessment of edema.

### Multimodality imaging assessments of MR-HIFU ablation

#### MRI

The soft tissue changes due to MR-HIFU ablation were assessed using MR images taken with a whole body SENSE rat coil (Rapid Biomedical, Germany) and a 3-T MRI scanner (Philips Healthcare, The Netherlands). T_2_-weighted MR images were acquired using a TSE sequence (TR = 8724 ms, TE = 28 ms, FOV = 70 × 70 × 25 mm^3^, voxel size = 0.35 × 0.35 × 0.70 mm^3^, NSA = 4) for evaluation of edema. T_1_-weighted FFE (TR = 20 ms, TE = 5.3 ms, FOV = 70 × 70 × 25 mm^3^, voxel size = 0.35 × 0.35 × 0.70 mm^3^, NSA = 4) images were obtained prior to and after injection of DOTAREM® at 0.2 mmol/kg body weight to assess tissue damage.

#### SPECT/CT

The area with high bone turnover following HIFU treatments was investigated using a high resolution small animal SPECT/CT scanner (NanoSPECT/CT®, Bioscan, USA) equipped with four detector heads and converging 9-pinhole collimators (pinhole diameter = 2.5 mm) in combination with ^99m^Tc-MDP. ^99m^Tc-MDP was injected at 76 ± 3 MBq. The CT of both femurs was acquired with 360 projections, 2-s exposure time per projection and a peak tube voltage of 65 kV, to provide anatomical reference. At 2 h after injection, SPECT images of both femurs were acquired with 32 projections and 200 s per projection. SPECT and CT images were reconstructed, and qualitative assessments of the SPECT images were done using InVivoScope software (Bioscan).

#### μCT

The bone micro-architecture of the treated and contralateral femur was acquired using a vivaCT 40 scanner (Scanco Medical AG, Switzerland). μCT images of the whole femur were acquired with a voxel size of 25 μm (45 kVp, 175 μA, 500 projections per 180°, 300-ms integration time).

### Three-point bending tests

**Fig. 3:**
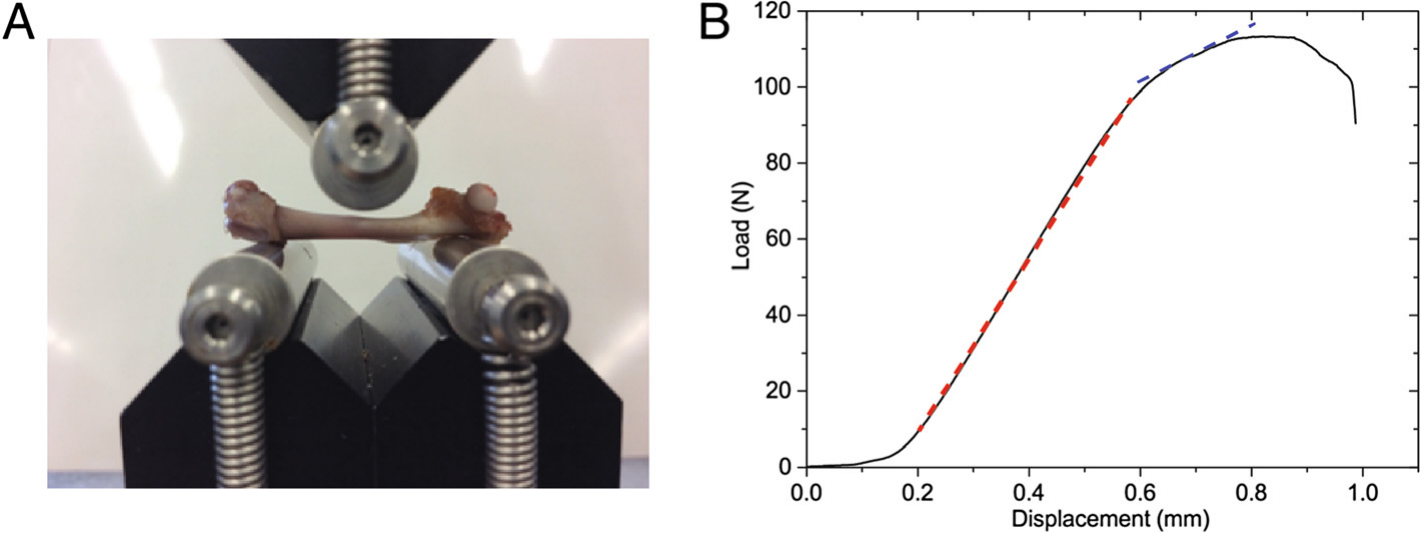
Three-point bending test. **a** The femurs were supported at the distal and proximal ends by two rolling pins while a load was applied in the mid-femoral shafts. **b** A representative load-displacement graph. Linear elastic range (*red dashed line*) and plastic range (*blue dashed line*)

A destructive three-point bending test was performed on both the HIFU-treated and contralateral control femurs (Universal testing machine Z 010/TN2S, Zwick, Ulm, Germany) to determine their elastic stiffness, ultimate load, and yield load, defined as the force at the changing point between the elastic and plastic ranges. The femurs were supported at the distal and proximal ends as depicted in Fig. 3a. Then, a load was applied in the mid-femoral shaft up to failure at a speed of 0.1 mm/min. The experimental bone elastic stiffness was defined as the slope of the linear elastic range from the obtained load-displacement graph (Fig. 3b). The changes in mechanical properties due to HIFU treatments were obtained by comparing results for the treated femurs with those of their respective contralateral control femurs at both time intervals. The time-dependent change in mechanical properties due to HIFU treatments was obtained by comparing the ratios of the HIFU-treated divided by the contralateral control bones at 7 days and 1 month post treatments.

### Micro-FE analyses of bone biomechanical properties

The μCT scans were rotated in such a way that their orientation corresponded to that in the three-point bending experiments. In order to reduce the total number of elements for the micro-FE analyses, the image resolution was reduced to 50 μm. Thereafter, images were thresholded using a single threshold of 500 per mille of the maximum possible value to segment solely the normally mineralized bone tissue. Then, the images were cropped to include only the region between the bottom rollers plus 0.5 mm on each side. The images were converted to micro-FE models using a voxel conversion technique [35]. Linear elastic material properties were assigned to all materials with a Young’s modulus of 10 GPa for the bone elements while the Poisson’s ratio was set to 0.3. Boundary conditions were chosen to represent the rollers’ support conditions at the bottom while a vertical displacement was prescribed at the location where the top roller was in contact with the bone. The bone stiffness was defined similarly to the three-point bending experiment. Finally, the ratio of the experimentally determined stiffness and the micro-FE-calculated stiffness was determined. After multiplying this ratio with the bone tissue value of 10 GPa prescribed in the micro-FE models, the actual tissue modulus can be back calculated [35]. All image processing and micro-FE simulations were done using IPL v5.16 (Scanco Medical AG, Brüttisellen, Switzerland).

### Histological analyses

The femurs were decalcified in 12.5 % ethylenediaminetetraacetic acid pH 7.4 solutions. Next, samples were processed for paraffin embedding. The femurs were sectioned at a 4-μm thickness using a microtome. Subsequently, the samples were machine-stained (Artisan^TM^ Link Pro, Dako, Belgium) with hematoxylin and eosin (H&E). Histological images were acquired at 20× magnification (ScanScope XT, Aperio, USA).

### Statistical analyses

The Wilcoxon signed rank test was used for all paired comparisons of the HIFU-treated versus the contralateral control bones in the three-point bending tests and micro-FE analyses. The two-sided *t* test was used to compare if the bone mechanical properties (elastic stiffness, ultimate load, yield load, and Young’s modulus) changed from 7 days to 1 month due to HIFU treatments, to investigate the changes in mechanical properties of bones due to growth from 7 days to 1 month, and to evaluate the differences in stiffness obtained from the three-point bending tests and micro-FE analyses. Analyses were performed using SPSS Statistics (v22, IBM, USA). Data are presented as the mean ± standard deviation (sd), with *P* < 0.05 considered significant.

## Results

### Multimodality imaging of MR-HIFU ablation

**Fig. 4:**
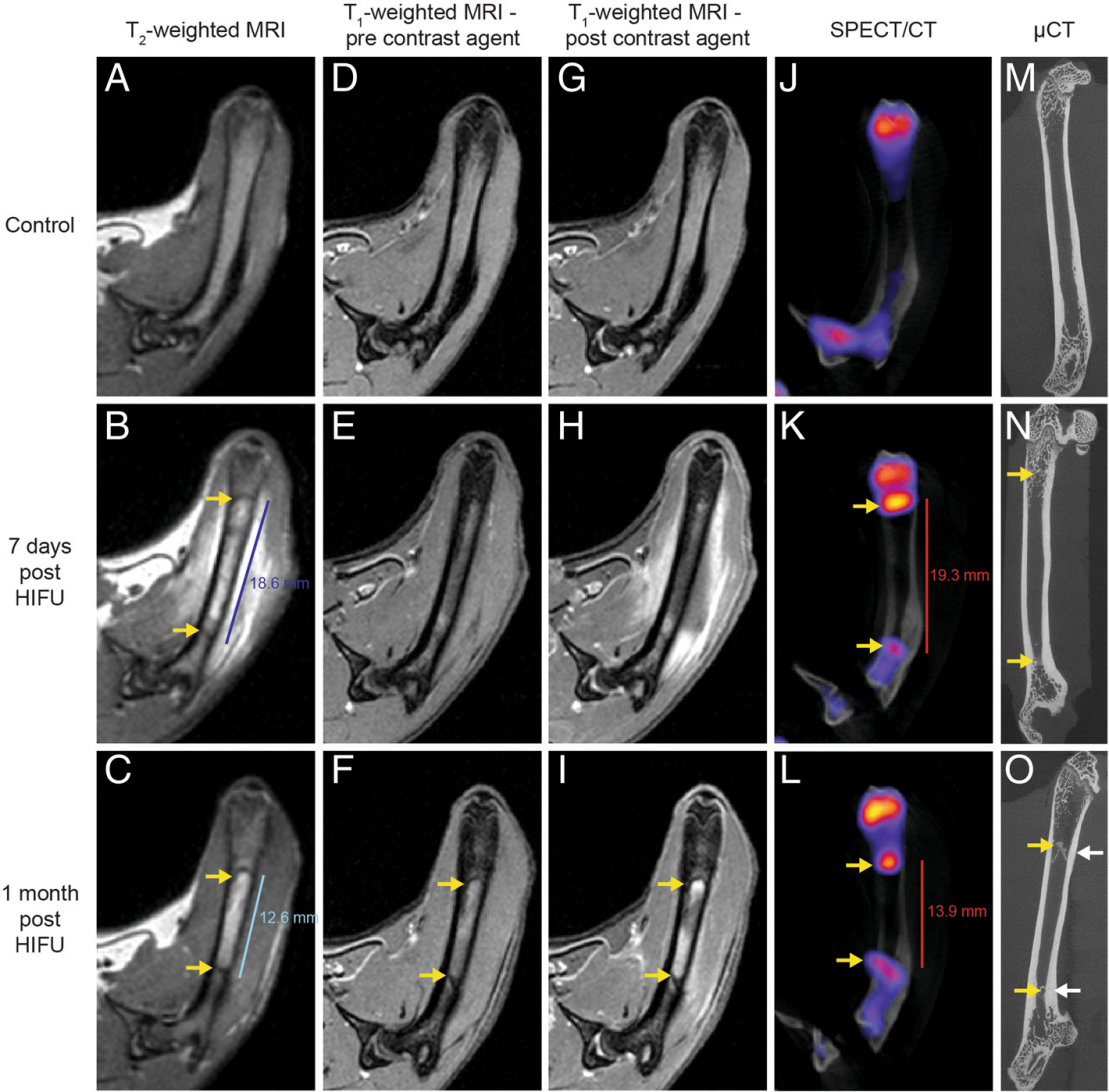
Multimodality imaging of MR-HIFU ablation. **a–c** *T*_*2*_ *-weighted MRI* of femurs before (**a**), at 7 days (**b**), and at 1 month (**c**) after ablations. **b** Edema was observed around the treated bone while mixed hypo- and hyper-intense signal was present in the intramedullary space. **b–c**, **f**, **i** *Yellow arrows* show lines with hypo-intense signal, which are similar to cortical bone, in the intramedullary space. **d–i** T_1_-weighted MRI prior to (**d–f**) and after (**g–i**) contrast agent injections. **h** Non-perfused region was observed around the treated bone after contrast agent administration. **i** Hypo-intense region in the intramedullary space suggest bone marrow damage. **j–l** *SPECT/CT* images before (**j**) and at 7 days (**k**) as well as 1 month (**l**) after HIFU ablation. **k–l** Areas with increased ^99m^Tc-MDP accumulation (*yellow arrows*) were detected within the intramedullary space and on the cortical surface. **m–o** *μCT* images of femurs before (**m**) and after (**n**–**o**) HIFU ablation. **n–o** Woven bone formation (*yellow arrow*) was observed in the intramedullary space at 7 days and 1 month post treatments. **o** Cortical thickening (*white arrows*) was present at 1 month after treatments

At 7 days after MR-HIFU ablation, treatment-associated edema, a region with hyper-intense signal around the bone compared to control in T_2_-weighted MR images, was observed (Fig. 4a, b). Within the intramedullary space, two lines with hypo-intense signal similar to cortical bone were found to flank the borders of the edema, while mixed hypo- and hyper-intense signals were present in the intramedullary space suggesting bone marrow damage (Fig. 4b). The corresponding T_1_-weighted MR images after injection of DOTAREM® showed comparable non-perfused region, marked by hypo-intense signal (Fig. 4d, e, g, h). SPECT/CT images at the same time point showed increased uptake of ^99m^Tc-MDP in the intramedullary space and on the surface of the cortical bone (Fig. 4j, k). These hotspots were also located at the periphery of the ablation zones. In line with MR and SPECT/CT images, woven bone formation was detected within the intramedullary space of the μCT images (Fig. 4m, n).

At 1 month after MR-HIFU ablation, edema around the treated femurs had resolved (Fig. 4c). Interestingly, the two lines with hypo-intense signal still remained, but the distance between these lines had shrunk from 18.6 to 12.6 mm (Fig. 4b, c). The T_1_-weighted images showed comparable hypo-intense signal lines within the bone marrow (Fig. 4f, i). Following injection of contrast agents, the non-perfused region in the surrounding tissue had diminished (Fig. 4i). The intramedullary space showed regions of hypo-intense signal, indicating bone marrow damage (Fig. 4i). In addition, similar to MR images, the distance between the HIFU-induced hotspots in SPECT/CT images had decreased from 19.3 to 13.9 mm, confirming progression of bone modeling (Fig. 4k, l). Moreover, increased uptake of ^99m^Tc-MDP was observed on the cortical bone surface. As expected, visual inspection of the μCT images confirmed the presence of woven bone formation and cortical thickening (Fig. 4o).

### Three-point bending test

**Fig. 5:**
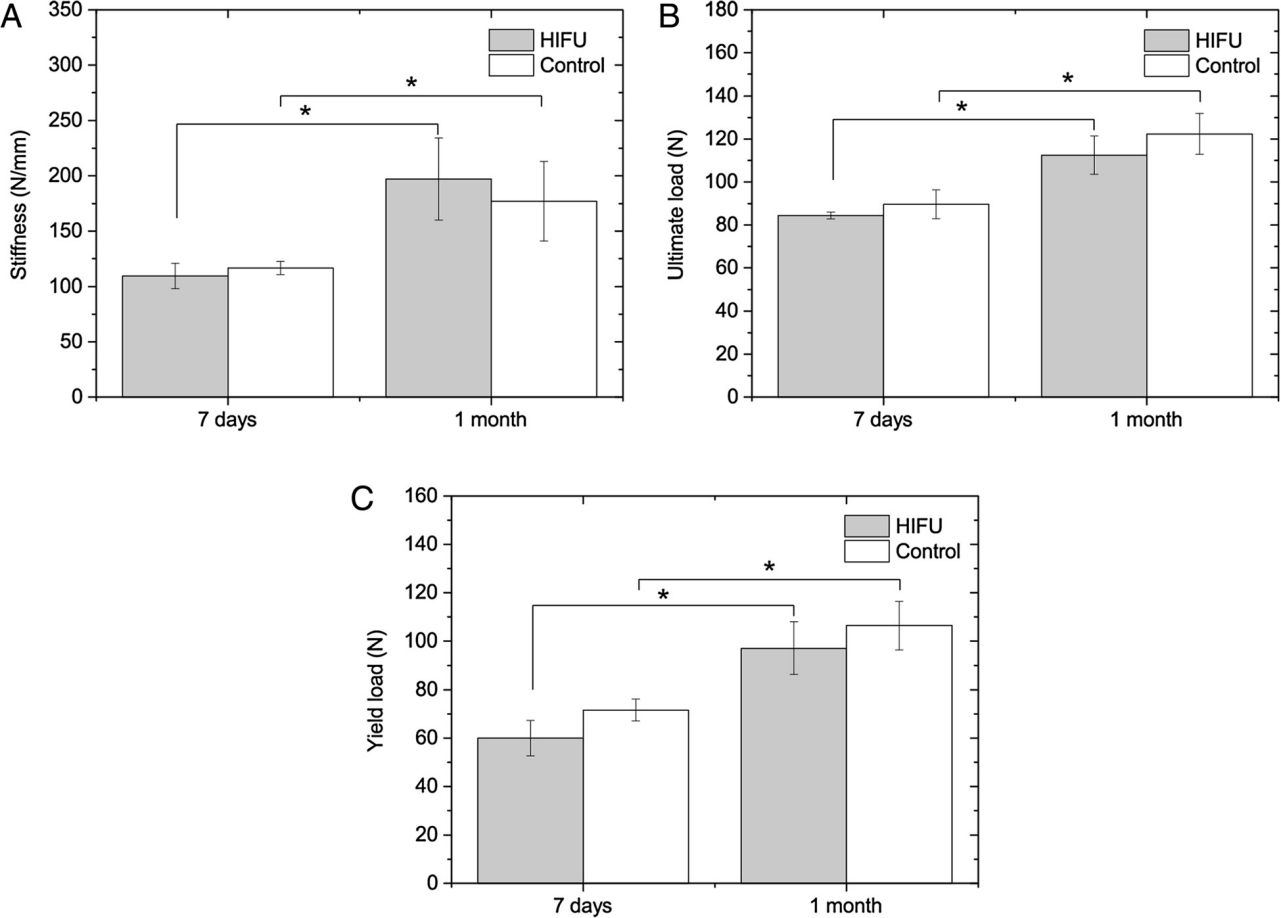
Mechanical properties of the HIFU-treated and contralateral control bones at 7 days and 1 month after treatments determined by three-point bending tests. **a** Elastic stiffness. **b** Ultimate load. **c** Yield load. *P < 0.05

The average elastic stiffness, ultimate load, and yield load of the HIFU-treated and contralateral control bones at both time intervals after ablations are shown in Fig. 5. At 7 days, the stiffness, ultimate load, and yield load were 6 ± 15 %, 6 ± 6 % and 16 ± 5 % less than those of the control bones, respectively. However, no significant differences were found between the treated and control bones [*P* = 0.29 (stiffness, ultimate load), 0.11 (yield load)]. Due to growth of the animals at 1-month time point, the mechanical properties of the treated and control bones were significantly higher (*P* < 0.05). At this time point, the stiffness of the treated bone was 13 ± 16 % higher than that of the control ones, whereas the yield and ultimate loads were 8 ± 5 % and 8 ± 4 % less, respectively. Similarly, no significant differences between the treated and control bones were noted [*P* = 0.29 (stiffness), 0.11 (ultimate load, yield load)]. From 7 days to 1 month, by comparing the ratio of the HIFU-treated divided by control bone, no significant changes in elastic stiffness (*P* = 0.60), ultimate load (*P* = 0.12), and yield load (*P* = 0.09) due to HIFU treatments were observed.

### Micro-FE analyses

**Fig. 6:**
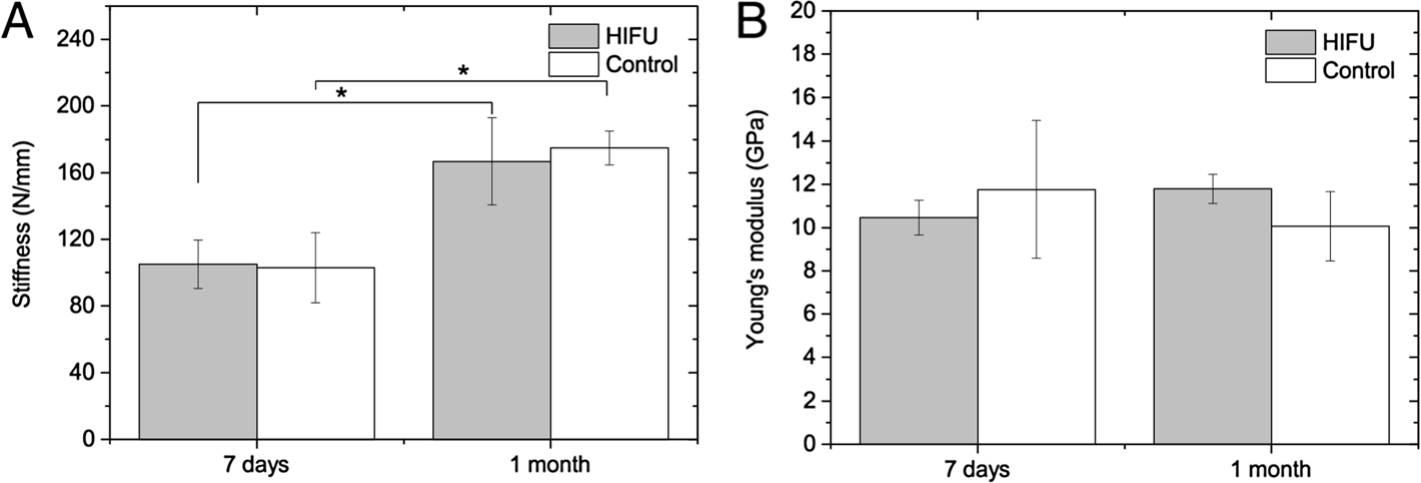
Mechanical properties of the HIFU-treated and contralateral control bones at 7 days and 1 month after treatments determined by micro-FE analyses. **a** Elastic stiffness. **b** Young’s modulus. *P < 0.05

The stiffness values measured in the three-point bending tests (Fig. 5) for the two time intervals were in good agreement with the predictions of the micro-FE analyses (Fig. 6). For the micro-FE calculated stiffness, no significant differences were found between the treated and control bones at 7 days (*P* = 0.59) and 1 month (*P* = 0.29) after treatments. In addition, there was a significant increase in stiffness over time due to the growth (*P* < 0.05). The calculation of the tissue Young’s moduli by comparing the experimental and micro-FE results revealed no significant differences between the treated and control bones at 7 days (*P* = 0.59) and 1 month (*P* = 0.11). Likewise, by comparing the ratio of the HIFU-treated divided by control bone, no significant differences due to HIFU treatments were found between the two time intervals for elastic stiffness (*P* = 0.49) and Young’s moduli (*P* = 0.22).

### Histological analyses

**Fig. 7:**
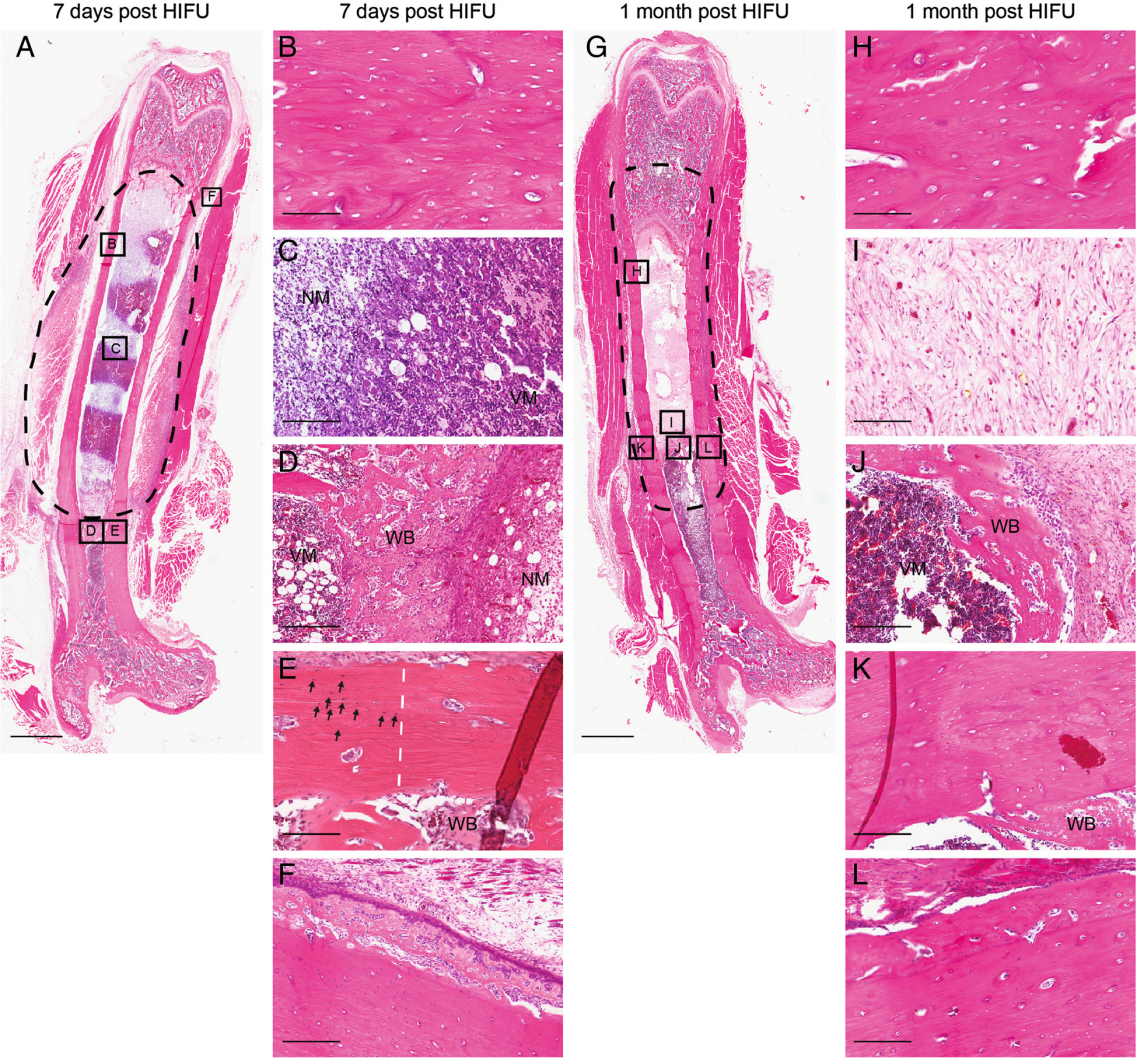
Histological analyses of bones following MR-HIFU ablations. **a** Whole femur at 7 days post treatment with ablation zone delineated with *dashed lines*. Ablation of the soft tissue surrounding the bone was observed. **b** Osteonecrosis of the cortical bone, marked by *empty lacunae*. **c** Bone marrow showed a mixture of viable and non-viable cells. **d** Woven bone (*WB*) lying in between the necrotic (*NM*) and viable marrow (*VM*) in the intramedullary space. **e** A sharp demarcation (*white dashed line*) between ablated and viable osteocytes (*arrows*) was observed at the border of the ablated area. **f** Sub-periosteal woven bone formation at the edge of the ablation zone. **g** Whole femur at 1 month post treatment with ablation zone delineated with *dashed lines*. A smaller region of soft tissue damage was observed around the bone. **h** Osteonecrosis of the cortical bone, marked by *empty lacunae*. **i** Fibrovascular structures and immature collagenous matrix in intramedullary space. **j** Woven bone flanked by viable marrow (*VM*) and fibrosis. **k** Necrotic cortical bone adjacent to the woven bone in the intramedullary space. **l** Sub-periosteal woven bone with higher calcification, leading to thickening of the cortical bone. Scale bar = 2.5 mm (**a**, **g**), 100 μm (**b**, **h**), 150 μm (**c**, **i**), 250 μm (**d**–**f**, **j**–**l**)

At 7 days after MR-HIFU treatments, ablation of bones and the surrounding soft tissue was observed (Fig. 7a). Within the ablation zone, osteonecrosis of the cortical bone, marked by empty lacunae, was noted (Fig. 7b). In line with the heterogeneous hypo- and hyper-intense signal in T_2_-weighted MR image at 7 days post ablation (Fig. 4b), the intramedullary space exhibited a mixture of viable and non-viable bone marrow (Fig. 7c). At the borders of the ablated zone, woven bone formation was observed in the intramedullary space (Fig. 7d). Moreover, at the same locations, a sharp demarcation between the ablated and viable osteocytes was detected (Fig. 7e). Sub-periosteal woven bone formation was also present at the periphery of the ablated area (Fig. 7f).

At 1 month after MR-HIFU treatments, the surrounding soft tissue had started healing leading to a smaller area of soft tissue damage, which was in line with the MR images (Figs. 4 and 7g), while the cortical bone remained necrotic (Fig. 7h). A part of the necrotic marrow had been replaced by an immature collagenous matrix made up of fibrovascular structures, an indication of bone marrow repair (Fig. 7i). Similar to the 7-day time point, woven bone was present in the intramedullary space (Fig. 7j). Consistent with the imaging results (Fig. 4), the distance between the woven bones in the intramedullary space had decreased (Fig. 7g). Although the woven bone in the intramedullary space had moved inward to allow for the return of the viable marrow cells, the adjacent cortical bone remained necrotic (Fig. 7k), suggesting a faster bone marrow healing process compared to the cortical bone. In agreement with the μCT results (Fig. 4o), the woven bone on the periosteal surface was more calcified, thereby contributing to cortical thickening (Fig. 7l).

## Discussion

Bone pain as a result of tumor growth affects 75 % of patients with advanced cancer [36]. These patients experience not only severe pain but also reduced quality of life. As radiation therapy (RT) is a suboptimal pain rescue treatment approach for localized painful lesions, development of alternative treatments for patients who do not respond to RT is crucial [37, 38]. MR-HIFU ablation is a promising technique for radiation refractory patients. Through understanding the bone functional changes and bone modeling induced by MR-HIFU, this technique can be applied in a safer and more effective manner. Herein, using multimodality imaging techniques, three-point bending tests, micro-FE, and histological analyses, the effects of MR-HIFU ablation on weight-bearing bone were investigated.

In this study, by means of multimodality imaging and histological analyses, we show that MR-HIFU ablations led to necrosis of bone marrow, cortical bone, and the surrounding soft tissue at 7 days post treatments, which is in agreement with previously published results [26–28]. At 1 month after treatment, soft tissue healing was noted, marked by a reduction in amount of soft tissue damage surrounding the bones. Moreover, as illustrated by H&E images, bone healing was evident through formation of woven bone and fibrovascular structures along with an immature collagenous matrix in the intramedullary space at the investigated time points. These bone healing processes are similar to those that occurred in response to mechanically induced bone marrow ablation in long bones [33, 34, 39]. With time, resorption of the woven bone will ensue to allow re-establishment of the hematopoietic and fat cells in the bone marrow cavity [33], a phenomenon also present in this study. In addition, sub-periosteal reaction adjacent to ablation sites, an indicator of intramembranous bone regeneration, was detected and resembles the results observed by others [26, 28, 34].

Besides that, data from the three-point bending tests show that the elastic stiffness, ultimate load, and yield load of the HIFU-treated bones did not significantly differ from the contralateral control bones at 7 days and 1 month after treatments, which were in agreement with previously published results on non-weight-bearing bones [29]. As expected, between the 7 days and 1 month time intervals, significant differences were observed in mechanical properties, which was due to the presence of normal growth. However, changes in mechanical properties due to MR-HIFU ablations alone were not discerned between the investigated time intervals. By combining the experimental and micro-FE results, the Young’s moduli of bone tissue were obtained to examine potential changes at bone tissue level, which could be caused, for instance, by micro-cracks or collagen damage. Since no significant differences in the tissue Young’s moduli were found at both time intervals following treatments, we conclude that this is not the case. In line with previous observations, no HIFU-induced fractures were observed following treatments [17, 29]. One possible explanation for the lack of any significant difference in bone stiffness and strength between the treated and control bones is the fact that necrotic bone at the investigated time intervals has the same mechanical properties as healthy bone. In our study, we deliberately assessed the effect of HIFU ablation using healthy bones to first understand HIFU effects as long as the bone structure and stability are not compromised by the presence of lesions. In treatment of bone lesions, particularly those with osteolytic phenotype, the effects of MR-HIFU would differ as these tumor-bearing bones are more prone to formation of fractures, but HIFU may also cause re-mineralization of the lesion. Therefore, future studies should be performed on lesion-bearing bones using the tools presented here.

## Conclusions

In conclusion, using MRI, SPECT/CT, μCT, and histological analyses, we demonstrated that MR-HIFU ablations induced bone damage at the cellular level, thereby triggering bone repair and modeling. However, based on three-point bending tests and micro-FE simulations, the resulting cellular damage did not compromise the mechanical function of the bone or cause micro-cracks at the bone tissue level.
